# Supplementary material for: Interfacial chemistry-driven reaction dynamics and resultant microstructural evolution in lithium-based all-solid-state batteries
Source: Nat Commun. 2025 Oct 3;16:8838. doi: 10.1038/s41467-025-63959-1 (PMC12494980; doi:10.1038/s41467-025-63959-1)
Supplement: Supplementary file 1 — Supplementary Information [file 41467_2025_63959_MOESM1_ESM.pdf]

SUPPLEMENTARY INFORMATION

Interfacial Chemistry-Driven Reaction Dynamics and Resultant Microstructural Evolution in Lithium-based All-Solid-State Batteries

Chanhyun Park[†], Jingyu Choi[†], Seojoung Park[†], Hyeong-Jong Kim, Yunseo Kim, Gukhyun Lim, Juho Lee, Eunryeol Lee, Sugeun Jo, Jiwon Kim, Jinsoo Kim, Jun Lim, Taeseok Kim, Jihyun Hong[*], Donghyuk Kim[*], and Sung-Kyun Jung[*]

Table of Contents

- Part. S1: Synthesis and characterization of coating layer of LiDFP NCM
  - Supplementary Figs. 1, 2, 3, 4, 5 and Tables 1, 2
- Part. S2: Investigation of chemical degradation with surface modification
  - Supplementary Figs. 6, 7, 8 and Table 3
- Part. S3: Electrochemical performance with mitigated chemical reactivity
  - Supplementary Figs. 9, 10, 11
- Part. S4: Quantification of charge heterogeneity with different surface chemistry
  - Supplementary Figs. 12, 13, 14, 15, 16, 17
- Part S5: Digital twin 3D reconstruction of FIB–SEM images for cathode composites
  - Supplementary Figs. 18, 19, 20, 21, 22, 23, 24, 25, 26, 27 and Notes. 1, 2
- Part. S6: Quantification of mechanical degradation at the particle level in ASSBs
  - Supplementary Figs. 28, 29, 30, 31, 32, 33, 34, 35 and Tables 4, 5

Part S1. Synthesis and characterization of coating layer of LiDFP NCM

The uniformity of the coating layer on the cathode surface is crucial in maintaining a consistent chemical composition, which is vital for the stability and performance of ASSBs. Understanding the precise composition of the coating material is fundamental for advancing material research and development. To verify the homogeneity and intended composition of the interfacial layer after the mechano-fusion coating process, we conducted SEM (Supplementary Fig. 1), STEM (Supplementary Fig. 2a) and ToF-SIMS (Supplementary Figs. 2b, c) analyses. These techniques confirmed the uniform distribution and chemical integrity of the coating layer. To gain a deeper understanding of the mechanical degradation behavior caused by chemical degradation at the interfaces in all-solid-state batteries, we analyzed the differences in mechanical behavior resulting from interfacial modification. Cross-sectional SEM images of cathode composites were taken immediately after cell fabrication to compare the microstructural evolution in a fresh state, prior to electrochemical operation (Supplementary Fig. 3). Additionally, to eliminate the interference of complex interfacial degradation caused by oxidation reactions of the coating material, we evaluated the electrochemical stability of the coating material within the operating voltage range (Supplementary Figs. 4 and 5). These findings demonstrate that LiDFP-based coating materials effectively modify the chemical reactivity at the interface, mitigating interfacial chemical degradation and enabling a deeper understanding of degradation behavior in all-solid-state batteries.

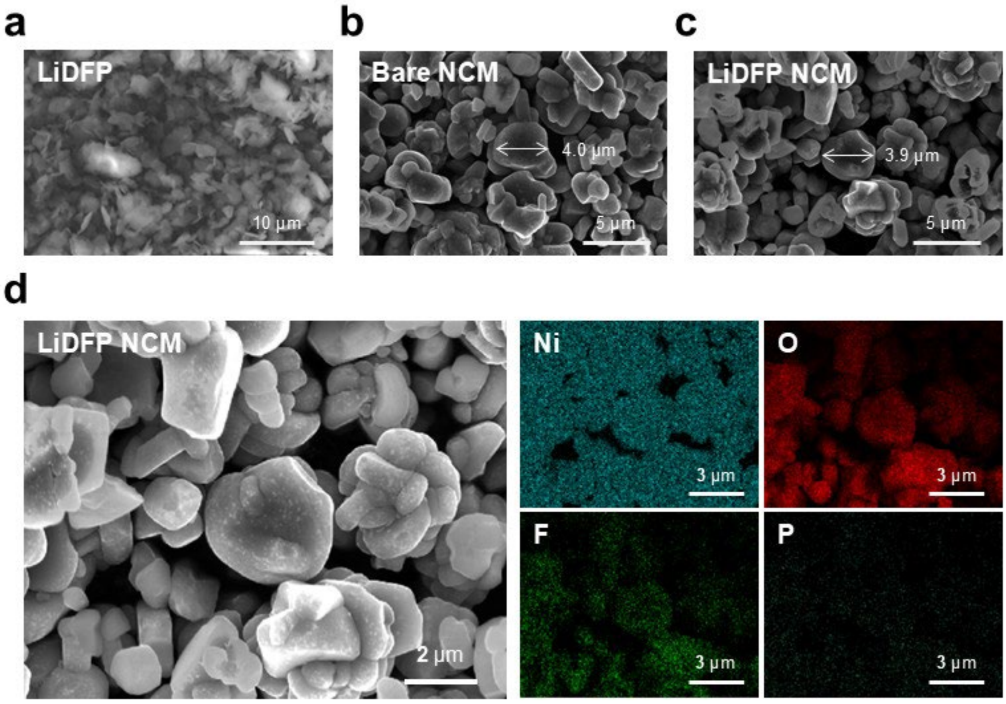

Supplementary Fig. 1. Top-view SEM images of a) Bare NCM, b) LiDFP NCM and c) LiDFP coating material. d) EDS mappings of LiDFP NCM, showing the distribution of Ni, O, F, P elements.

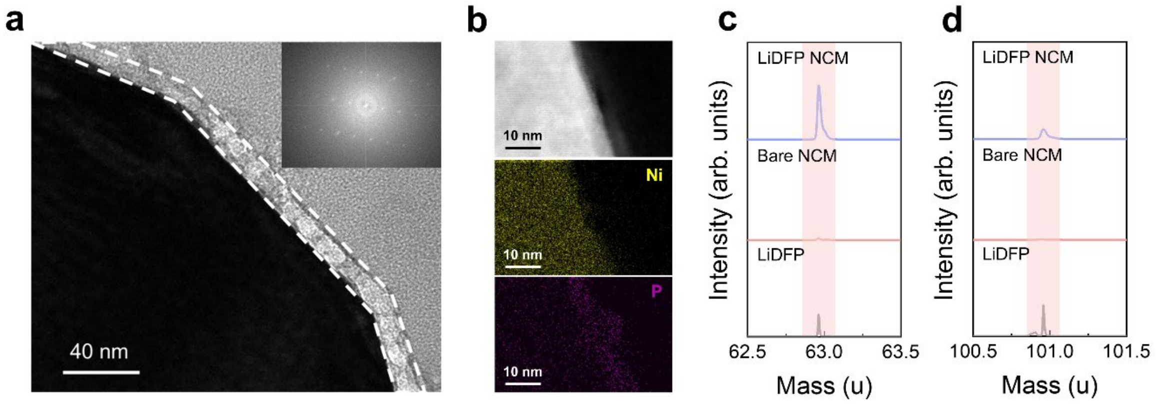

Supplementary Fig. 2. a) Cross-sectioned STEM image and corresponding FFT image of NCM surface coated with 0.3 wt% LiDFP, denoted as LiDFP NCM. b) Cross-sectioned STEM image and corresponding EDS mapping for Ni and P atoms. TOF-SIMS surface spectra of LiDFP additive, bare NCM, and LiDFP NCM of c) PO_2^- anion and d) $PO_2F_2^-$ anion.

Supplementary Table 1. Voltage, current, resistance, thickness, area, and electronic conductivity value for current–time curves of the cathode-SE composite (65:35 wt%) for both bare NCM and LiDFP NCM in a steel|NCM composite|steel ion blocking cell.

Cathode/SE (65/35)	$V_{composite}$ (V)	$I_{composite}$ (mA)	$R_{composite}$ (Ω)	$L_{composite}$ (cm)	$A_{composite}$ (cm^2)	**$\sigma_{composite, e}$ (S/cm)**
Bare NCM	0.2	0.000657	304414	0.02789	1.32665	**6.90 E-08**
LiDFP NCM	0.2	2.47E-05	8097166	0.02947	1.32665	**2.74E-09**

Supplementary Table 2. Voltage, current, resistance, thickness, area, and ionic conductivity value for current–time curves of the cathode-SE composite (65:35 wt%) for both bare NCM and LiDFP NCM in In/InLi|LPSCl|NCM composite|LPSCl|In/InLi electron blocking cell.

Cathode/SE (65/35)	V_{total} (V)	I_{total} (mA)	R_{total} (Ω)	$R_{composite}$ (Ω)	$L_{composite}$ (cm)	$A_{composite}$ (cm^2)	**$\sigma_{composite, i}$ (S/cm)**
Bare NCM	0.05	0.122866	406.9474	231.6386	0.02823	1.32665	**9.19 E-05**
LiDFP NCM	0.05	0.122549	408.0001	232.6913	0.02892	1.32665	**9.37 E-05**
LPSCl	0.05	0.285211	175.3088	175.3088	0.11014	1.32665	**4.74 E-04**

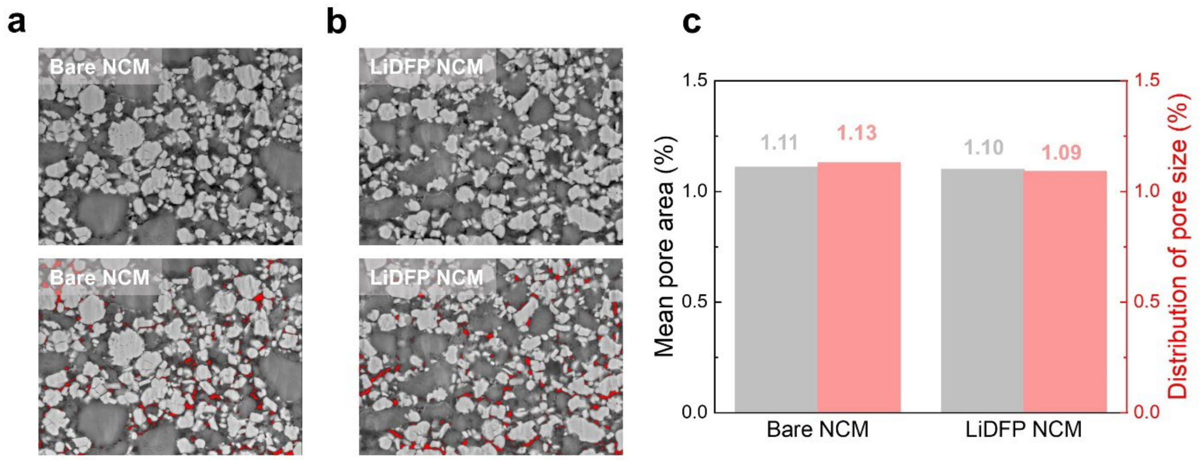

Supplementary Fig. 3. Cross-sectional SEM images of a) bare NCM composite and b) LiDFP NCM composite right after the cell fabrication and corresponding c) mean pore area ratio and distribution of pore for fresh cells. Red dots represent the pore at the cathode composite.

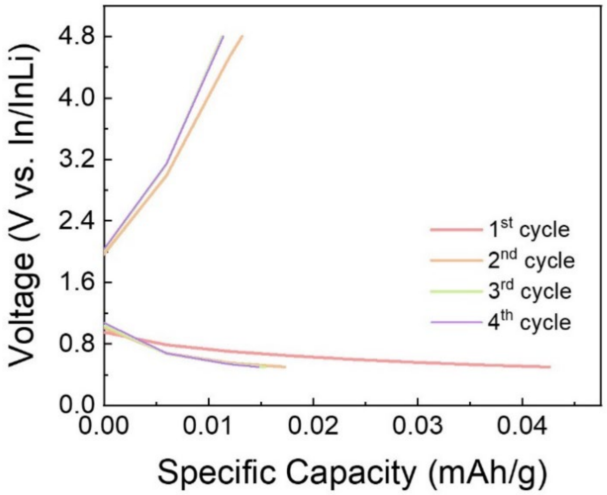

Supplementary Fig. 4. Voltage profiles of initial and substantial cycles of In/InLi|LPSCl|LiDFP NCM composite full cell.

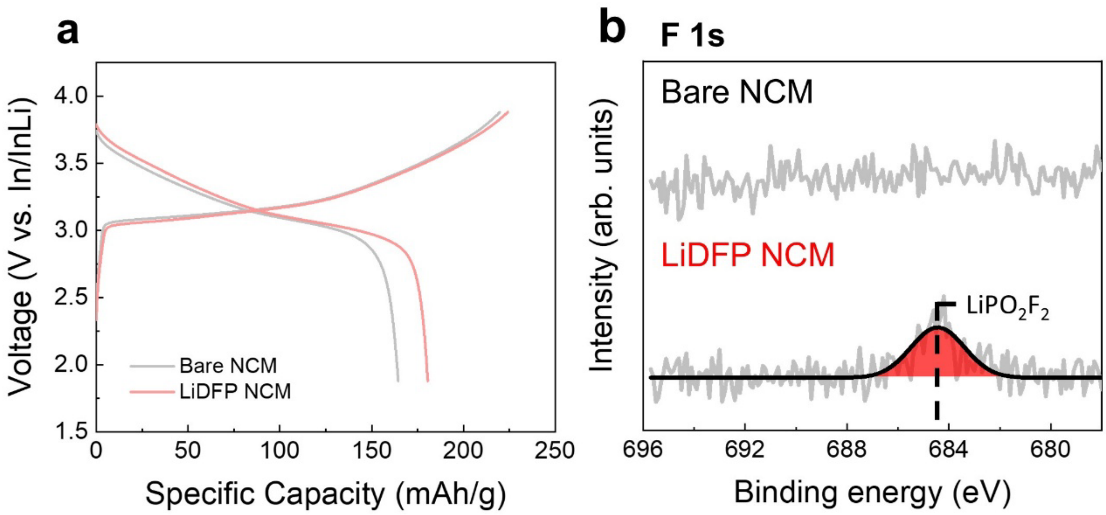

Supplementary Fig. 5. a) Voltage profiles during the initial cycle of In/InLi|LPSCl|NCM composite cells and corresponding b) F 1s XPS spectra of cathode composite after initial cycling of In/InLi|LPSCl|NCM composite full cells, within the voltage range of 1.88–3.88 V (*vs.* In/InLi) at C/10 (1C = 1.36 mA/cm^2) and 25 °C under a stack pressure of 13 MPa with 7.4mg/cm^2 active material mass loading.

Part S2. Investigation of chemical degradation kinetics with surface modification

To investigate the chronic chemical instability of sulfide solid electrolytes under high-voltage conditions and elucidate the role of coating materials, we conducted an analysis of the chemical degradation kinetics within a diffusion-controlled model, where the interfacial diffusion is restricted to ion transport across the interphase under OCV conditions (Supplementary Fig. 6). Utilizing distribution of relaxation times (DRT) analysis, we distinctly identified resistive components at varying frequencies: high frequency for the grain-boundary region of the cathode composite, mid-frequency for the cathode–SE interphase, and low frequency for the anode–SE interphase (Supplementary Figs. 7 and 8).[2,3] This approach enabled a comprehensive assessment of the chemical degradation kinetics and the protective efficacy of the coating material under high-voltage conditions.

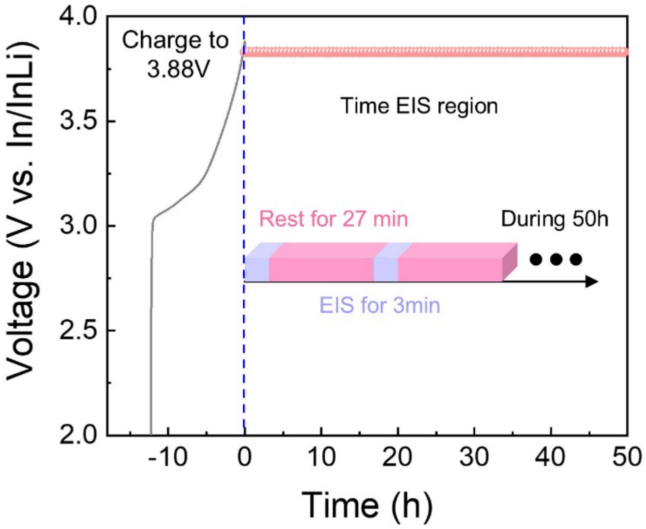

Supplementary Fig. 6. Representative voltage profile of a In/InLi|LPSCl|NCM composite full cell with 50-h rest after charging up to 3.88 V (*vs.* In/InLi) under C/10 (1C = 1.36 mA/cm^2) at 25 °C with a stack pressure of 13 MPa.

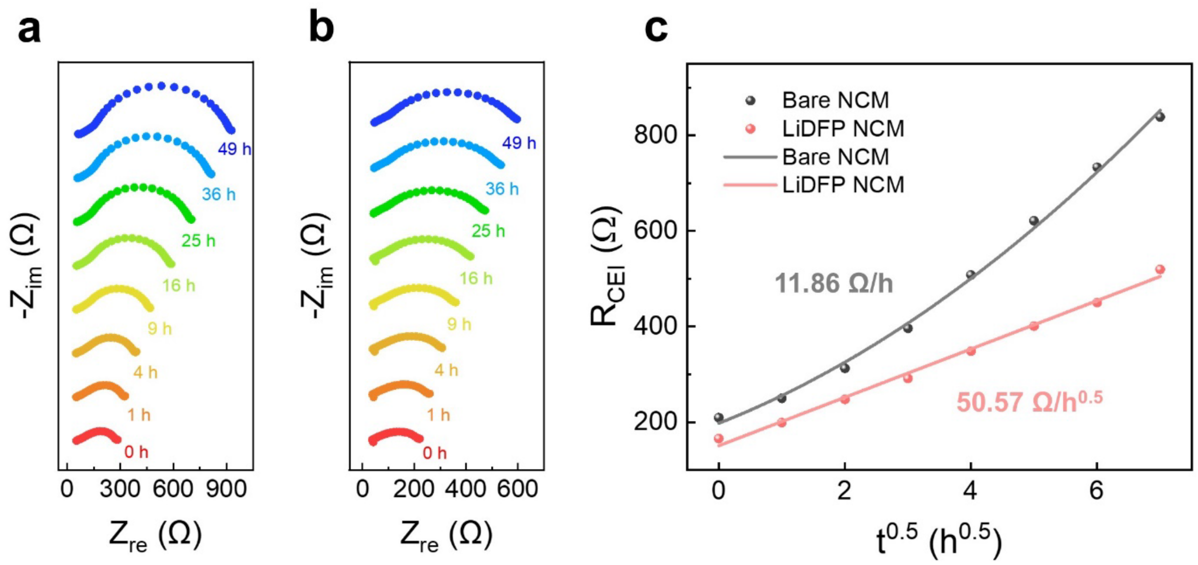

Supplementary Fig. 7. Nyquist plots of a) bare NCM and b) LiDFP NCM in In/InLi|LPSCl|NCM composite full cells after charging up to 3.88 V (*vs.* In/InLi) under C/10 (1C = 1.36 mA/cm^2) at 25 °C with a stack pressure of 13 MPa. c) Comparison of interface resistance R_{CEI} change as a function of the square root of time ($t^{0.5}$), highlighting differences between bare NCM and LiDFP NCM.

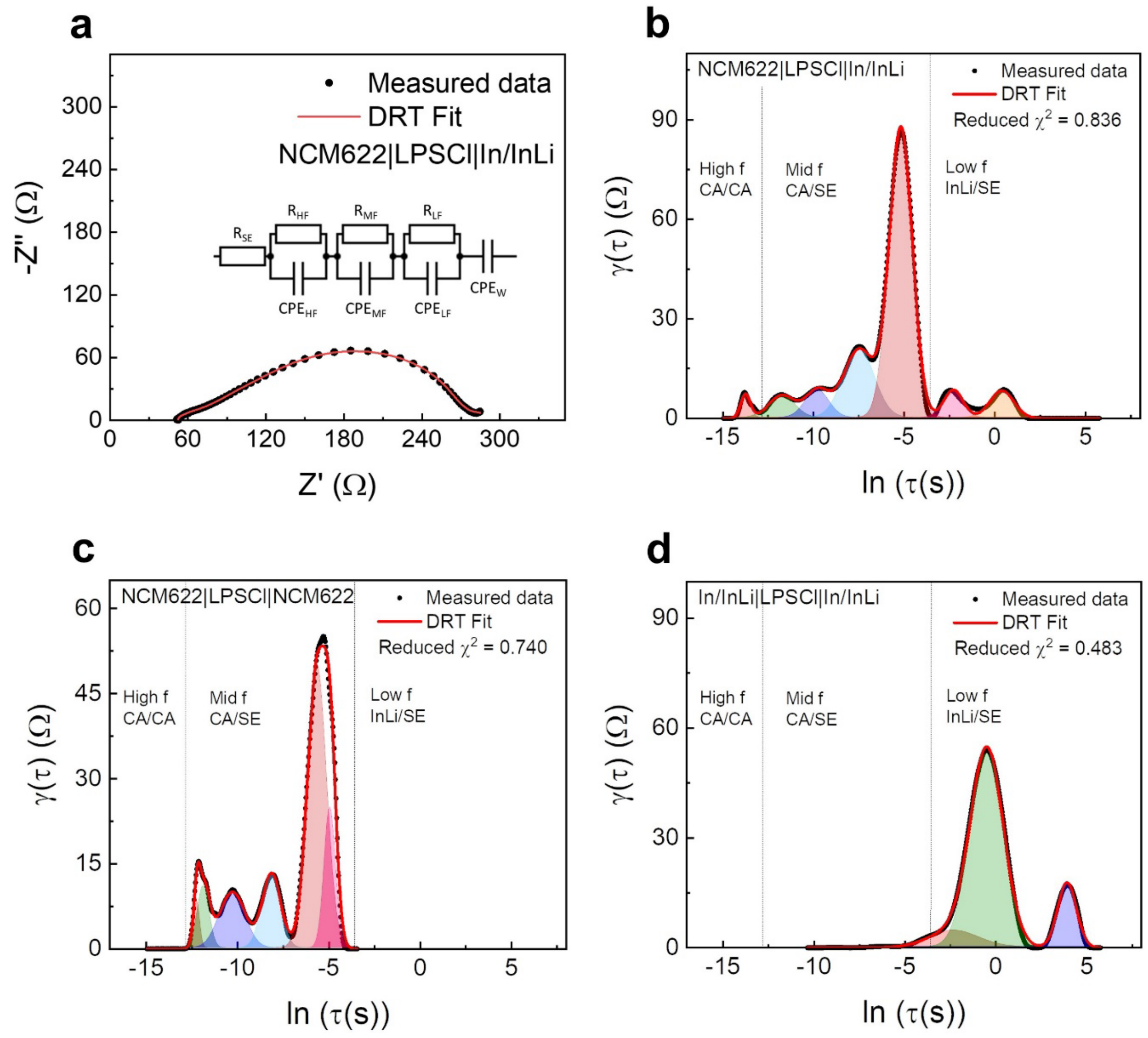

Supplementary Fig. 8. Representative a) Nyquist plot and DRT plot of a b) In/InLi|LPSCl|NCM composite full cell after charging up to 3.88 V (*vs.* In/InLi) under C/10 (1C = 1.36 mA/cm^2) at 25 °C with a stack pressure of 13 MPa. Representative DRT plot of c) In/InLi symmetric cell and d) cathode composite symmetric cell.

Supplementary Table 3. Symbols and denoted meanings for CA|SE interface resistance.

Symbol	Meaning
S	Contact area
$\overline{\sigma_{CEI}}$	Average ionic conductivity of CA|SE interface layer
V_m	Average molar volume of CA|SE interface layer
x	Number of moles of Li extracted from LPSCl
F	Faraday's constant
$\overline{\sigma_{Li^+}}$	Partial ionic conductivity of CA|SE interface layer
$\overline{\sigma_{e^-}}$	Partial electronic conductivity of CA|SE interface layer
μ_{Li}	Difference in the chemical potential of lithium across the interface layer
t	Resting time
$k,\ k'$	Rate constant

Part S3. Electrochemical performance with mitigated chemical reactivity

Unlike traditional LIBs that utilize liquid electrolytes, ASSBs form interfaces through solid–solid point contacts, necessitating external pressure during operation.[4] ASSB operation can be broadly categorized based on the applied pressure: low-pressure operation below 10 MPa, medium-pressure operation in the tens of MPa, and high-pressure operation reaching hundreds of MPa.[1] This study aims to compare the electrochemical characteristics of ASSBs under varying pressure environments, with a focus on medium-pressure operation at 13 MPa, as detailed in Part S3 (Supplementary Figs. 9 and 10). Reproducible electrochemical cycle performance was confirmed in Supplementary Fig. 11. The electrochemical evaluation under these conditions will provide fundamental insights into the intertwined interfacial chemo-mechanical effects on the electrochemical performance and stability of ASSBs across different operational pressures.

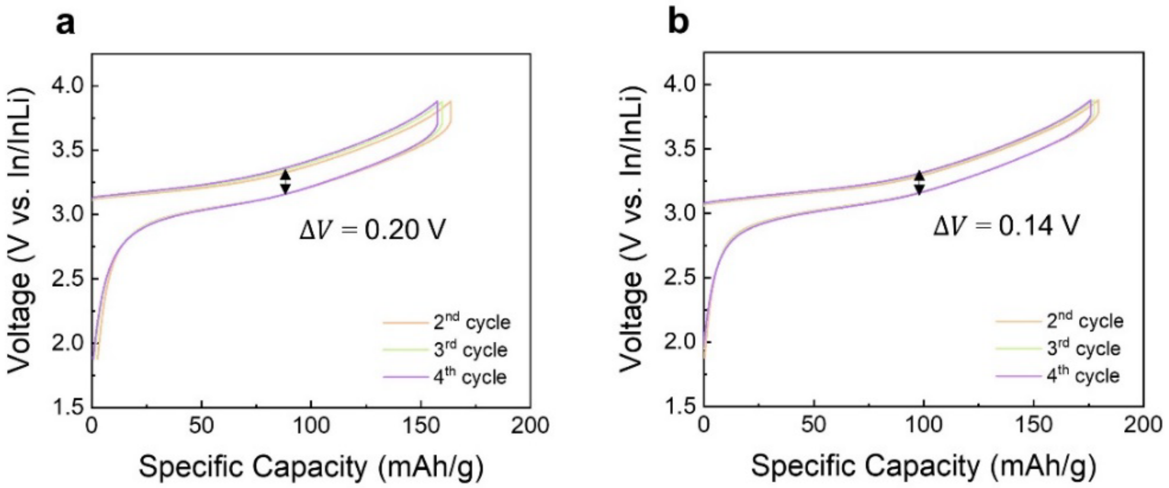

Supplementary Fig. 9. Voltage profiles of 2nd to 4th cycle of a) bare NCM and b) LiDFP NCM of In/InLi|LPSCl|NCM composite full cell in the range of 1.88–3.88 V (*vs.* In/InLi) under C/10 (1C = 1.36 mA/cm^2) at 25 °C with a stack pressure of 13 MPa with 7.4mg/cm^2 active material mass loading.

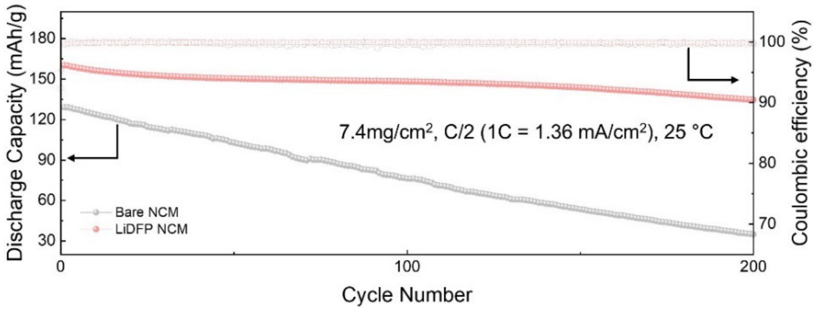

Supplementary Fig. 10. Comparison of long-term cycle performance between bare NCM and LiDFP NCM of In/InLi|LPSCl|NCM composite full cell in the range of 1.88–3.88 V (*vs.* In/InLi) under C/10 (1C = 1.36 mA/cm^2) at 25 °C with a stack pressure of 13 MPa with 7.4mg/cm^2 active material mass loading.

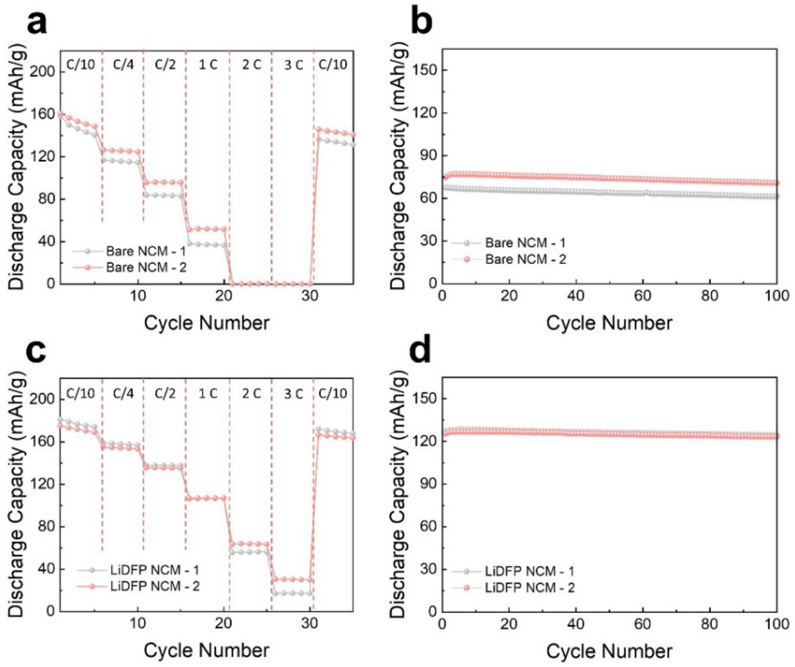

Supplementary Fig. 11. Reproducible electrochemical cycle performance of a, b) bare NCM and c, d) LiDFP NCM. Rate capability of a) bare NCM and c) LiDFP NCM at varying C-rates: C/10, C/4, C/2, 1C, 2C, 3C, and back to C/10 and substantial long-term cycle performance of b) bare NCM and d) LiDFP NCM at C/2 between bare NCM at 25 °C under a stack pressure of 120 MPa with two reproducible cells. Each cell labeled as '1' in the figure corresponds to the cell shown in Fig. 1e and f.

Part S4. Quantification of charge heterogeneity with different surface chemistry

To quantitatively assess the particle uniformity under conditions of interfacial chemical degradation at the micro-scale, advanced X-ray analytical techniques such as *in-situ* XRD (Supplementary Fig. 12) and TXM were employed. These techniques provide detailed insights into the structural and chemical changes occurring within cathode materials.

- *In-situ* XRD experiments for quantitative analysis:

The behavior of cathode particles, influenced by surface modifications, was explored by examining characteristic changes in the (003) peak of the layered structure under electrochemical conditions (Supplementary Fig. 13). To quantify the particle heterogeneity at each SOC, the peaks were deconvoluted to statistically define the mean and standard deviation, establishing the DOH, as shown in Supplementary Fig. 14. The *in-situ* synchrotron XRD data for the liquid electrolyte system were collected at beamline 6D of the Pohang Light Source (PLS), utilizing a 1 M $LiPF_6$ in mixed carbonate (EC:DMC = 1:1 vol%) electrolyte. The coin cells for *in-situ* XRD measurements were specially designed with holes on both sides of the cases, sealed with Kapton films as X-ray transparent windows. The weighted average of the peak positions was calculated to compare the mean peak positions between the ASSB and LIB systems, facilitating a comparative analysis of cathode behavior influenced by surface modification and battery system configuration (Supplementary Fig. 15). To examine the behavior of cathode particles affected by interfacial chemical degradation due to the low oxidative stability of sulfide-based SEs, a cathode composite comprising the cathode, SE, and carbon was mixed and stored in a glove box for 72 h. Subsequently, an ASSB cell was assembled for *in-situ* XRD analysis (Supplementary Fig. 16).

- Ex-situ TXM with liquid-electrolyte-based LIBs:

To precisely understand the oxidation states of Ni in ASSBs during electrochemical

evaluation, ex-situ TXM analysis was conducted at SOC intervals of 20. This approach ensured a detailed examination of the oxidation state changes of Ni as the ASSB cell underwent cycling. Ex-situ TXM data were collected at beamline 7C of the PLS using a 1 M $LiPF_6$ in mixed carbonate (EC:DMC = 1:1 vol%) electrolyte with in-house beamline software.[5,6] To establish a linear correlation between the photon energy and changes in the oxidation state of Ni, the averaged peak position energy in XANES of the cathode particles was plotted at every SOC increment of 20, corresponding to a 55.2 mAh/g change in capacity (Supplementary Fig. 17). Energy-dependent images were acquired across the Ni K-edge energy range at 1eV intervals with an exposure time of 0.3 s for each image, and aligned using the in-house beamline software.

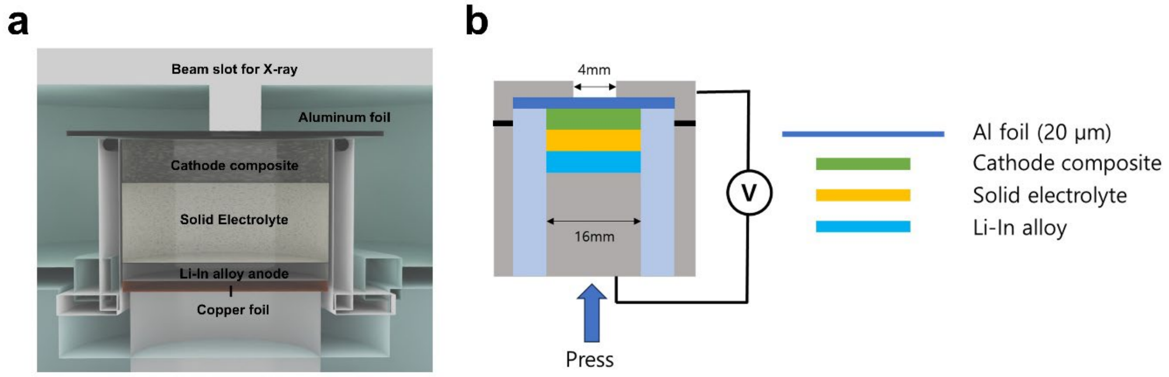

Supplementary Fig. 12. a) Cross-sectional images and b) schematic diagram for electrochemical operation of *in-situ* XRD module for ASSBs.

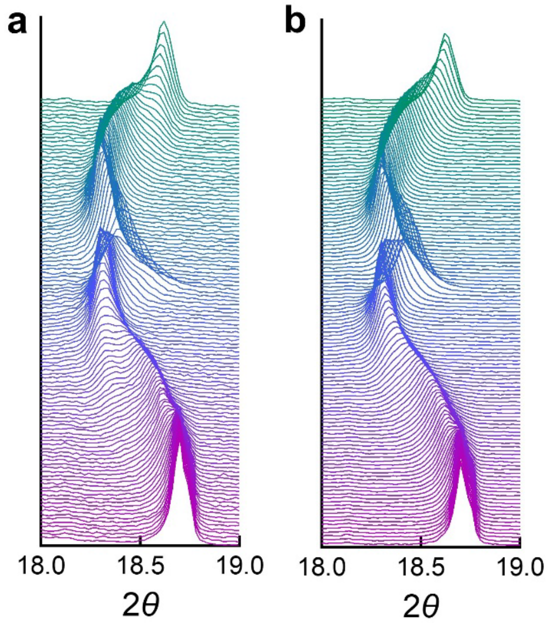

Supplementary Fig. 13. *In-situ* XRD spectra of a) bare NCM and b) LiDFP NCM at 18°–19° operated in the range of 1.88–3.88 V (*vs.* In/InLi) under a current density of 0.48 mA/cm^2 at 25 °C with a stack pressure of 13 MPa.

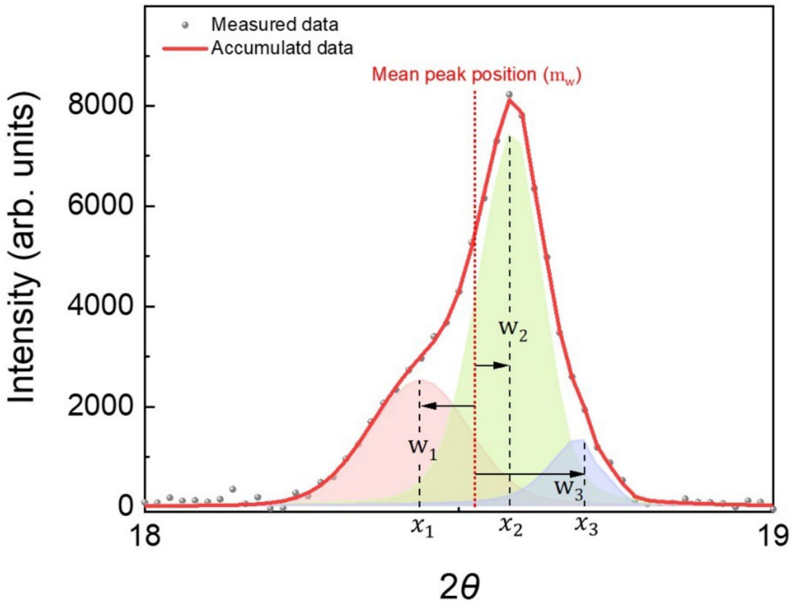

Supplementary Fig. 14. Representative peak deconvolution plot of a In/InLi|LPSCl|NCM composite full cell during *in-situ* XRD analysis.

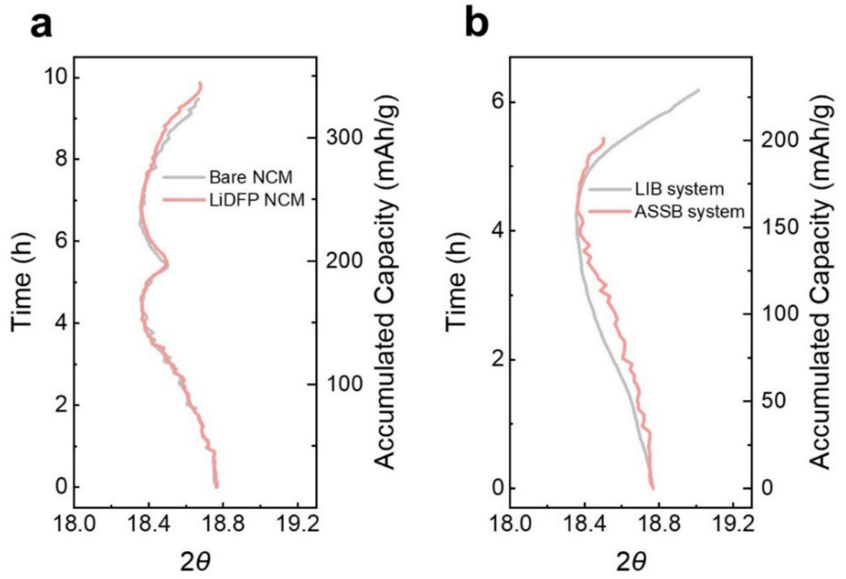

Supplementary Fig. 15. a) Mean peak position changes for bare NCM and LiDFP NCM within the ASSB system. b) Comparative analysis of mean peak position changes between LIB and ASSB systems.

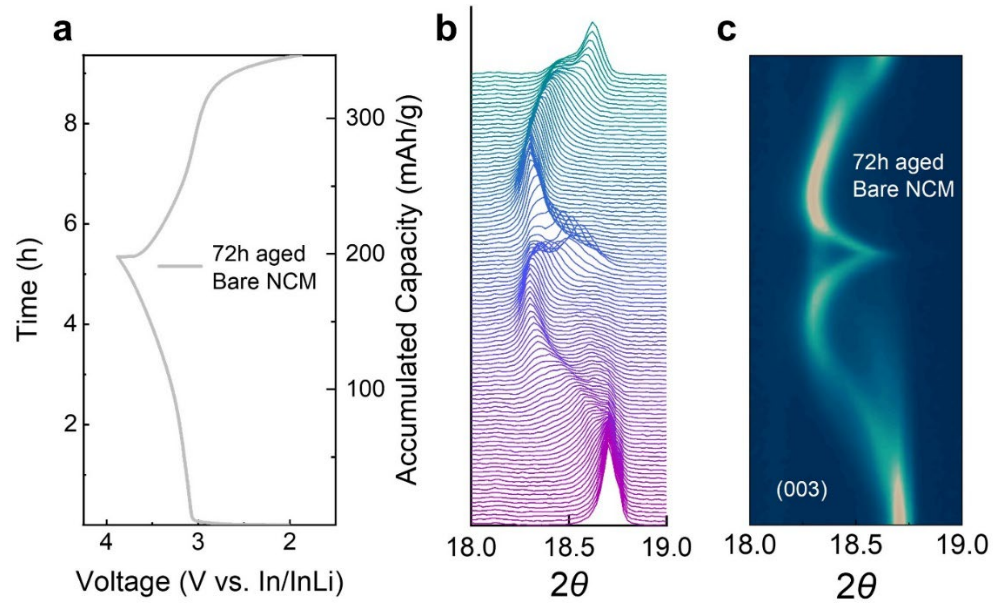

Supplementary Fig. 16. a) Voltage profile of 72h aged bare NCM and corresponding *in-situ* XRD b) spectra and c) contour plot of the diffraction peak evolution of the (003) planes in the range of 1.88–3.88 V (*vs.* In/InLi) under a current density of 0.48 mA/cm^2 at 25 °C with a stack pressure of 13 MPa with 7.4mg/cm^2 active material mass loading.

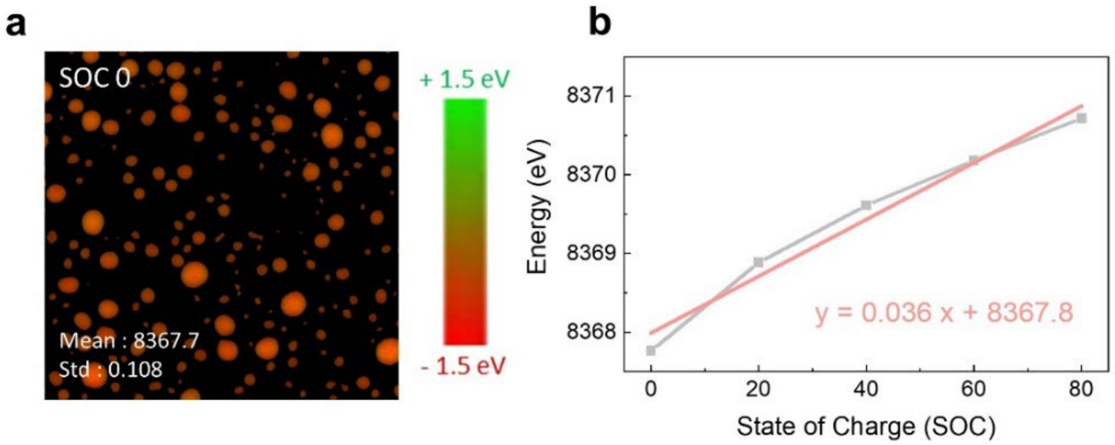

Supplementary Fig. 17. a) Ex-situ TXM–XANES mapping of pristine bare NCM and b) linear plot of SOC-dependent averaged peak position energy in XANES.

Part S5. Digital twin 3D reconstruction of FIB–SEM images for cathode composites

Before segmenting the cathode composite components, a wavelet-FFT function was applied to mitigate the curtaining effect (Supplementary Fig. 18).[7] To delineate the cathode, SE, carbon, and voids within the cathode composites of ASSBs, a convolutional neural network (CNN)-based U-net algorithm, typically utilized in biomedical image segmentation, was adapted. This advanced technique allowed for the precise segmentation of each component based on gray values of the studied images (Supplementary Figs. 19 and 20). This approach allowed for precise identification and separation of the individual components within the cathode composite.[8] To construct the 3D digital twin of NCM and its adjacent pores, each cross-sectional object was detected from segmented FIB–SEM images employing contour techniques in OpenCV. The contour function describes a curve connecting all continuous points where the intensity equals a given threshold. Each contour object of NCM and pores in segmented FIB–SEM images was extracted using a threshold criterion of 200 and 0, respectively, and the parameters were set to RETR_EXTERNAL mode and CHAIN_APPROX_NONE method (Supplementary Note. ML1 and Supplementary Fig. 21). To selectively isolate pores surrounding NCM, a (3, 3, 1)-dimension filter window was designed. This specialized window centers and screens each coordinate of NCM contours, enabling direct evaluation of neighboring pixels around the center point (Supplementary Fig. 22). If pore contours intersect with this window, these pores are filtered as adjacent ones. The detected contours of 2D cross-sectional NCM and adjacent pores were assigned unique identifiers, represented by an expression index. These identifiers, along with information such as the z-axis vertical positions and contour coordinates, were integrated into a graph structure, serving as the foundational data for developing the 3D digital twin system (Supplementary Figs. 23 and 24).

To achieve 3D objectification of NCM and adjacent pores within the digital twin space, linking the contours across cross-sections of the same NCM particles detected in each frame is essential. This paper presents an ML-based iterative template matching system (Supplementary Figs. 25 and 26), specifically developed for this purpose. Initially, virtual images, depicting a single NCM contour drawn on a black empty space that occupies the minimum size circumscribed to the contour, were created corresponding to the number of contours in the target FIB–SEM image. Each virtual image is designated as a template according to the NCM index order and used to screen the investigation contour map formed by dimensionally aligning with the target template shape and concatenating each virtual image from the subsequent FIB–SEM data. By calculating the correlations between the target template and investigation contour map using the TM_COEFF_NORMED parameter and manually curating to remove outliers, the NCM indexes with the highest correlations were connected for 3D objectification and assigned unique identifiers, represented by expression IDs (Supplementary Note. ML2). Utilizing graph data comprising NCM IDs, their corresponding 2D indexes, and frame numbers, pore contours can be classified based on adjacency to NCM IDs (Supplementary Fig. 27). Furthermore, the classified pore indexes around specific NCM were clustered based on the criterion that center-point distances were within 4 pixels, enabling 3D pore objectification. This objectification-based digital twin system enables interactive visualization of NCM and adjacent pores, along with numerical morphology analysis based on assigned IDs, thereby paving the way for comprehensive CA|SE interface analysis.

To comprehensively analyze the effect of LiDFP at the CA|SE interface, this study aimed to quantify and assess the morphological characteristics of the interface reconstructed in 3D digital twin space, as described in Figs. 3f, g. The graph network data format employed in this study to objectify NCM and its adjacent pores offers the advantage of retaining all relevant information, including the z-axis vertical position, (x,y) coordinates, 2D indices, and 3D IDs.

The surface area of the 3D objective and the volumetric information were calculated by summing the perimeters of the 2D contours, computed using the 'cv.arcLength()' function, and by summing the inner areas of the 2D contours, computed using 'cv.moments()' function, respectively. Both functions were provided by the OpenCV library. Additionally, the contact area between NCM and adjacent pores was calculated by screening the contour coordinates of NCM with those of the neighboring pores. NCM side coordinates within a distance of less than 2 pixels were defined as adjacent. The linear regression and Pearson correlation calculation used for trend analysis in Figs. 3f, g were performed using the 'linear_model()' function from the sklearn package and the 'corr()' function from pandas, respectively.

Supplementary Note. ML1. Approaches to extracting cross-sectional contour objects from segmented FIB–SEM images

This section presents a detailed description of the process for extracting the contours of NCM and its adjacent pores from FIB–SEM cross-section images.

1.1 Data processing for contour extraction of NCM

To objectify individual NCM particles in 2D images by detecting contours with uniform intensity, the segmented FIB–SEM images were first converted into grayscale and subsequently binarized. The contour function ‘cv.findContours()’ utilized morphological transformations through the ‘cv.morphologyEX()’ function, employing the opening operation (erosion followed by dilation) with a 10×10 kernel composed entirely of ones. Furthermore, the retrieval mode was set to ‘cv.RETR_EXTERNAL’, and the contour approximation method was defined as ‘cv.CHAIN_APPROX_NONE’. Among the detected boundary points, contours were excluded based on two criteria: (1) those containing fewer than 30 points, which are potentially ambiguous due to their insufficient size, and (2) those cut off by the edges of the image, which do not reveal their complete shape, as illustrated by the blue outlines in Supplementary Fig. 21.

1.2 Data processing for contour extraction of pores

To detect pore contours, the FIB–SEM images underwent an inversion process due to an issue where the segmented process mapped the pore areas to intensity 0, thereby objectifying the regions surrounding the pores rather than the pores themselves. In the inverted images, the contour detection was performed using the same methodology as for the NCM contours, with the exclusion criterion adjusted to exclude contours with fewer than 25 points.

Supplementary Note. ML2. Methodologies for calculating correlations in the template matching system

To calculate the correlations between the target NCM template and the investigation contour map, the 'cv.matchTemplate()' function from OpenCV was used, employing the 'TM_COEFF_NORMED' method, which returns a correlation value of 1 for a perfect match, −1 for a perfect mismatch, and 0 for no correlation.

In this process, the virtual image in the (N)-*th* frame, corresponding to the target NCM index, scans the investigation contour map of the (N+1)-*th* frame to identify the linkage candidate NCM with the highest correlation that is adjacent along the z-axis to the target NCM in the N-*th* frame. This procedure is iteratively repeated by updating the target virtual image to the selected NCM in the (N+1)-*th* frame and linking the NCM indices to facilitate 3D objectification. If no corresponding NCM is identified with a maximum correlation threshold of 0.4, it is presumed that the detection process for the specific NCM particle across cross-sections is complete, and the results are reviewed through manual curation (Supplementary Fig. 25).

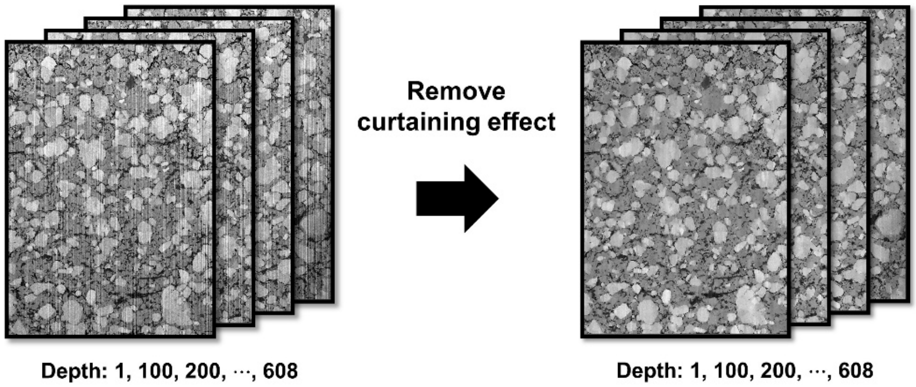

Supplementary Fig. 18. FIB–SEM stacking images before and after removing curtaining effect.

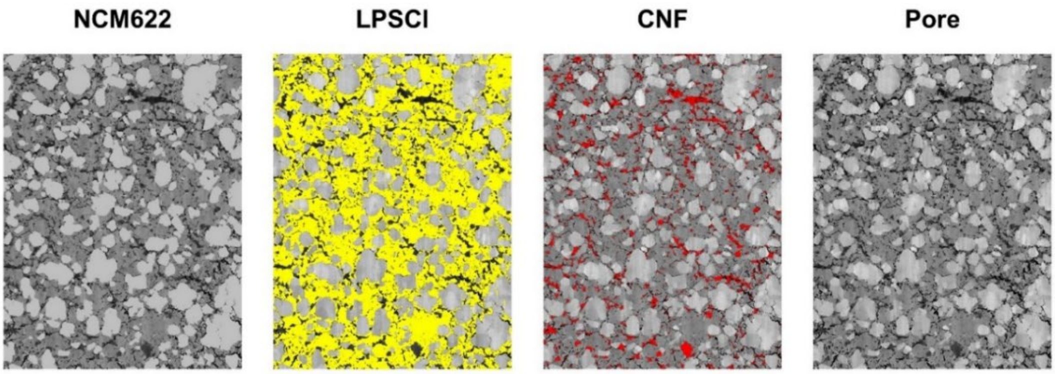

Supplementary Fig. 19. Segmented images for all components; gray-NMC, yellow-LPSCl, red-void, and black-carbon.

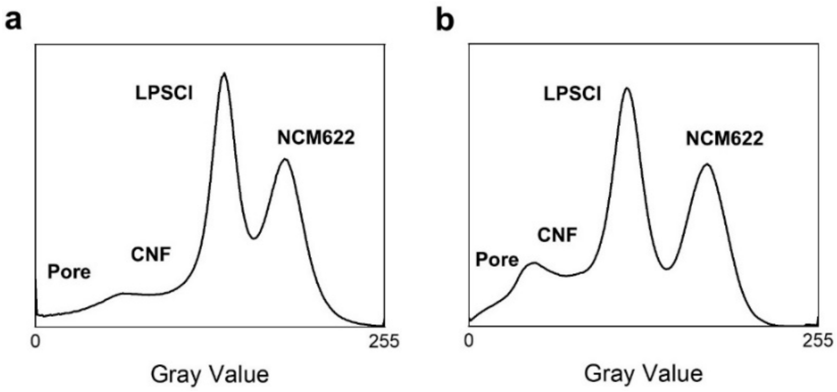

Supplementary Fig. 20. Gray value spectrum of a) bare NCM and b) LiDFP NCM.

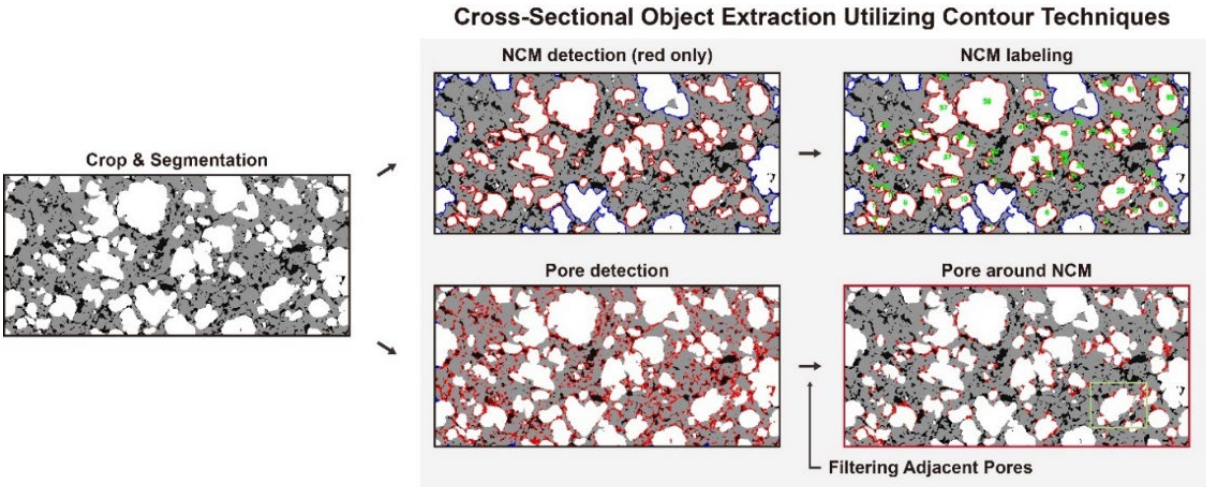

Supplementary Fig. 21. Extracted images of cross-sectional NCMs and pores using contour techniques in OpenCV. Detected NCMs and pores with incomplete shapes due to image boundaries (indicated by blue lines) and pores not adjacent to NCMs are filtered out.

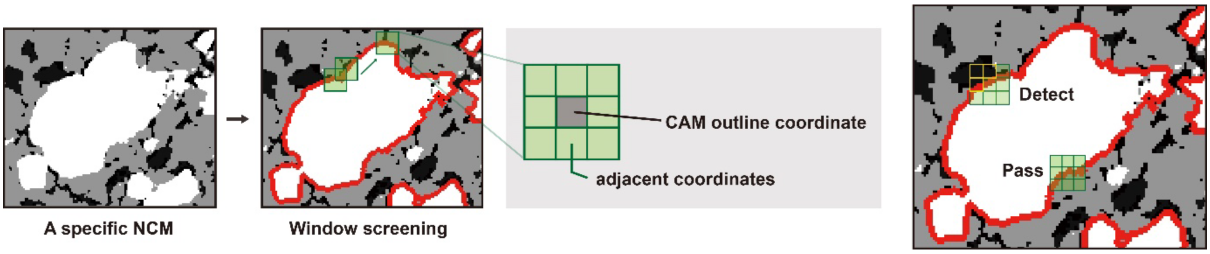

Supplementary Fig. 22. Schematic representation of filtering NCM-adjacent pores using (3, 3, 1)-dimension filter, which centers on each coordinate of NCM contours and screens neighboring pixel information.

a.

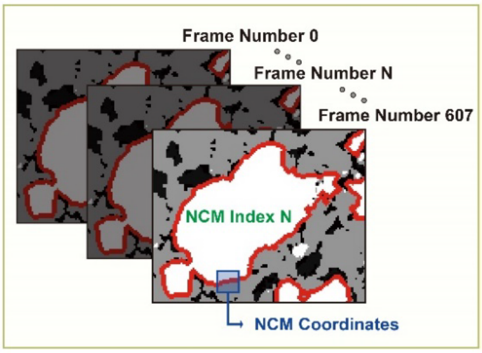

b.

Type	# Frame	NCM Index	Coordinates
Bare	0	0	[[[562, 1163]], [[561, 1164]], [[561, 1165]], ...
Bare	0	1	[[[137, 1160]], [[136, 1161]], [[135, 1162]], ...
Bare	0	2	[[[438, 1159]], [[437, 1160]], [[436, 1160]], ...
Bare	0	3	[[[104, 1152]], [[103, 1153]], [[102, 1154]], ...
Bare	0	4	[[[769, 1145]], [[768, 1146]], [[767, 1146]], ...
Bare	...	...	...
Bare	607	196	[[[480, 24]], [[480, 25]], [[479, 26]], [[478,...
Bare	607	197	[[[154, 14]], [[154, 15]], [[154, 16]], [[154,...
Bare	607	198	[[[335, 11]], [[334, 12]], [[333, 12]], [[332,...
Bare	607	199	[[[77, 9]], [[76, 10]], [[75, 10]], [[74, 10]]...
Bare	607	200	[[[734, 3]], [[733, 4]], [[732, 4]], [[731, 5]...

Supplementary Fig. 23. Unique identifiers allocated to detected 2D cross-sectional NCMs, denoted by an expression index. a) Graphical representation illustrating the assignment of NCM index. b) Tabular representation of the graph structure depicting NCM identifiers, corresponding frame information, and their coordinates, serving as the fundamental data for 3D objectification.

a.

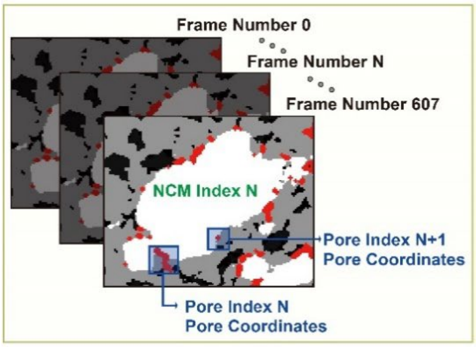

b.

Type	# Frame	NCM Index	Pore Index	Pore Coordinates
Bare	0	0	[]	[]
Bare	0	1	[0]	[[[133, 1156], [133, 1157], [133, 1158], ...
Bare	0	2	[1]	[[[455, 1152], [454, 1153], [453, 1154], ...
Bare	0	3	[2]	[[[116, 1166], [115, 1167], [114, 1168], ...
Bare	0	4	[]	[]
Bare	...	...	...	...
Bare	607	196	[213]	[[[486, 15], [486, 16], [485, 17], [484, 18],...
Bare	607	197	[]	[]
Bare	607	198	[214]	[[[355, 13], [355, 14], [355, 15], [356, ...
Bare	607	199	[215]	[[[62, 13], [61, 14], [60, 14], [59, 15], ...
Bare	607	200	[216]	[[[719, 27], [718, 28], [717, 29], [716, 30],...

Supplementary Fig. 24. Unique identifiers allocated to detected 2D cross-sectional NCM-adjacent pores, denoted by an expression index. a) Graphical representation illustrating the assignment of NCM adjacent pores. b) Tabular representation of the graph structure depicting NCM indices, adjacent pore identifiers, corresponding frame information, and the pore coordinates, serving as the fundamental data for 3D objectification.

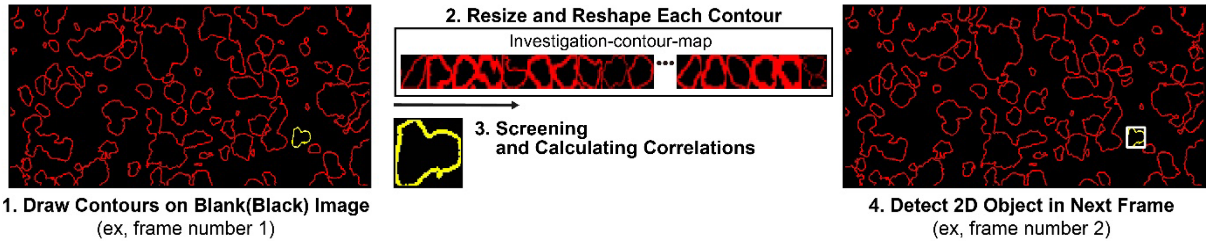

Supplementary Fig. 25. Flow diagram illustrating the iterative template matching system utilized to link contours across cross-sections of the same NCM particles in each frame, achieving a 3D digital twin system.

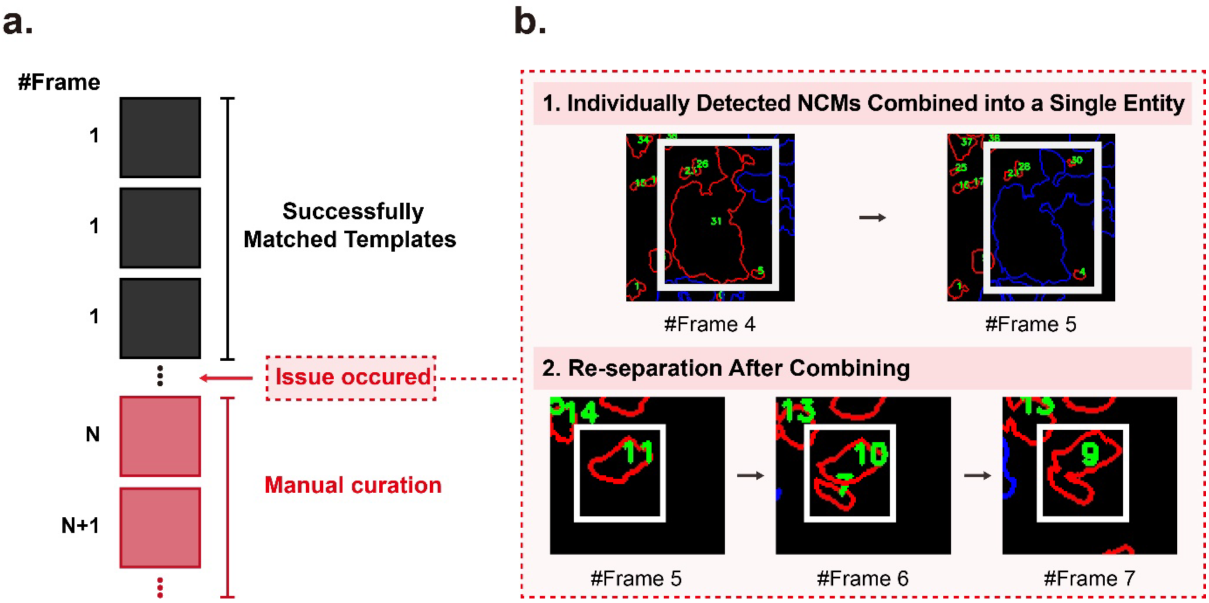

Supplementary Fig. 26. Schematic representation of handling outliers occurred in 3D objectification process. a) Structural diagram combining the template matching system with manual curation for handling outliers. b) Two illustrative examples of atypical outliers that can occur during the template matching process.

a.

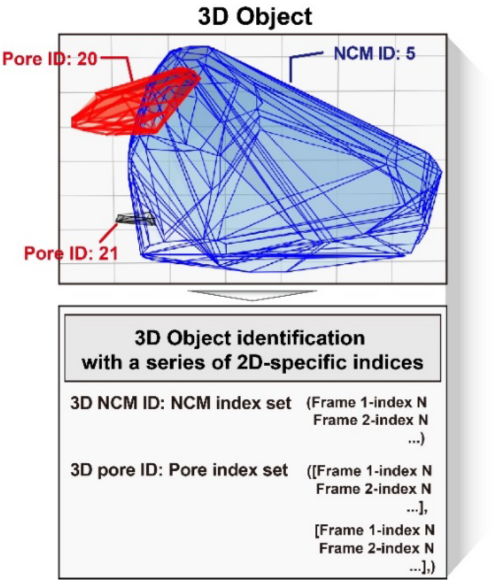

b.

Type	NCM 3D ID	#Frame for NCM	NCM 2D Index	Pore 3D ID	#Frame for Pore	Pore 2D Index
Bare	0	[100, 101, ...]	[11, 12, 8, ...]	[]	[]	[]
Bare	1	[102, 103, ...]	[25, 28, 28, ...]	[2, 2, 3, 4, ...]	[110, 111, ...]	[50, 26, 31, ...]
Bare	2	[104, 105, ...]	[102, 102, ...]	[21, 21]	[106, 112]	[113, 88]
Bare	3	[105, 106, ...]	[168, 164, ...]	[22, 23, 23 ...]	[105, 106, ...]	[163, 154, ...]
Bare	4	[105, 106, ...]	[64, 63, 68, ...]	[24, 24, 25, ...]	[130, 131, ...]	[63, 70, 47, ...]
Bare	···	···	···	···	···	···
Bare	495	[9, 10, 11, ...]	[105, 102, ...]	[]	[]	[]
Bare	496	[9, 10, 11, ...]	[141, 140, ...]	[]	[]	[]
Bare	497	[9, 10, 11, ...]	[151, 149, ...]	[3420, 3420, ...]	[24, 26, 27, ...]	[163, 137, ...]
Bare	498	[9, 10, 11, ...]	[171, 169, ...]	[3425, 3425, ...]	[9, 9, 10, ...]	[203, 204, ...]
Bare	499	[9, 10, 11, ...]	[18, 17, 17, ...]	[3444, 3445, ...]	[11, 11, 12, ...]	[20, 21, 20, ...]

Supplementary Fig. 27. Unique identifiers assigned to 3D objectified NCM and its adjacent pores, denoted by an expression ID. a) Graphical representation illustrating the 3D object identification and the data structure linking 3D IDs and 2D indices of both NCM and its adjacent pores. b) Tabular representation of the graph structure depicting 3D IDs, alongside comprehensive frame-specific data, including 2D indices for both NCM and its adjacent pores.

Part S6. Quantification of mechanical degradation at the particle level in ASSBs

Prior to the 3D quantification, we first addressed the limitations inherent in the 2D analysis (Supplementary Fig. 28). In quantification, each cathode particle was assigned a unique index, allowing for the quantification of key parameters such as the surface area and volume. Additionally, we systematically quantified the contact loss area, defined as the contact loss area with pores, along with the surface area, volume, and number of pores surrounding these particles. This approach enabled a comparative analysis of the pore characteristics induced by changes in the volume of the cathode particles. Such a methodology provides deeper insights into how microstructural changes at the particle level affect the overall battery performance (Supplementary Figs. 29, 31, and 34). To investigate electron and ion percolation within the cathode composite, which comprises an electron-conducting cathode and carbon alongside an ion-conducting electrolyte, a 3D current density map of the composite was generated. The electronic and ionic current density map was calculated using Ohm's law ($\boldsymbol{J} = \frac{\varepsilon}{\tau^2}\boldsymbol{\sigma}\boldsymbol{\nabla}\boldsymbol{E}$) and implemented as $\Delta V = 1$ V under Dirichlet boundary conditions, and this calculation was implemented using the explicit jump (EJ) solver of ConductoDict module in GeoDict2024 (Supplementary Fig. 30). In conjunction with GITT analysis, *in-situ* XRD was employed to determine the requisite time for ensuring the thermodynamic equilibrium of cathode particles. This approach facilitates a comprehensive understanding of lithium-transport dynamics within the composite, and quantifying contact loss through this method enables precise identification of active surface area for relative lithium transport comparison within the cathode composite (Supplementary Figs. 32 and 33).[9,10] Reproducible electrochemical cycle performance was confirmed in Supplementary Fig. 35.

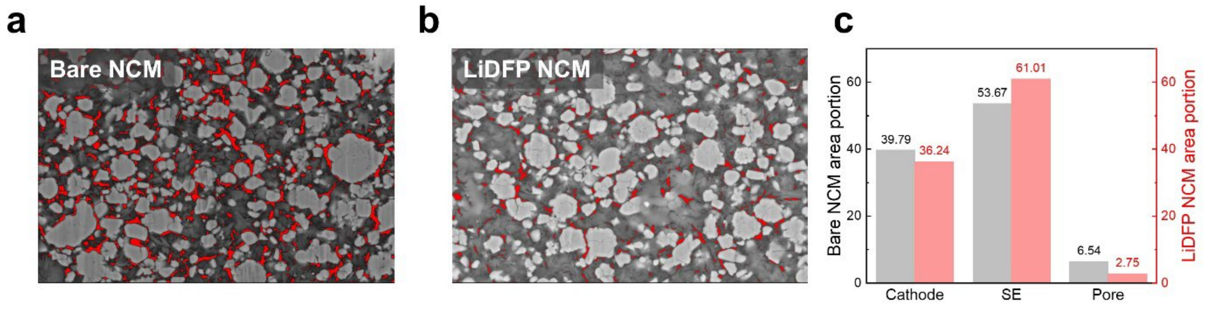

Supplementary Fig. 28. Cross-sectional SEM images of a) bare NCM and b) LiDFP NCM after precycling; the red regions indicate pore area and the corresponding c) area portion of cathode, SE, and pore for each SEM image.

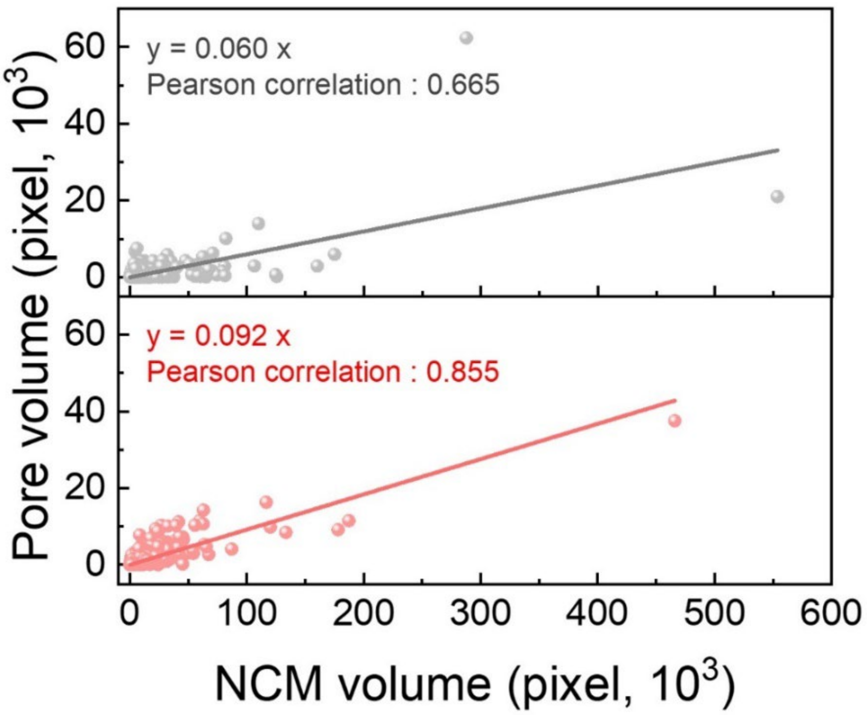

Supplementary Fig. 29. Comparison of cathode volume versus pore volume for labeled cathode particles, both bare NCM and LiDFP NCM.

Supplementary Table 4. Symbols and denoted meanings for ionic (or electronic) current density.

Symbol	Meaning
J	Ionic (or electronic) current density (A/m^2)
ε	Volume fraction of ionic (or electronic) conductive material
τ	Tortuosity
σ	Intrinsic ionic (or electronic) conductivity (S/m)
∇E	Electrical potential difference in Dirichlet boundary condition (V)

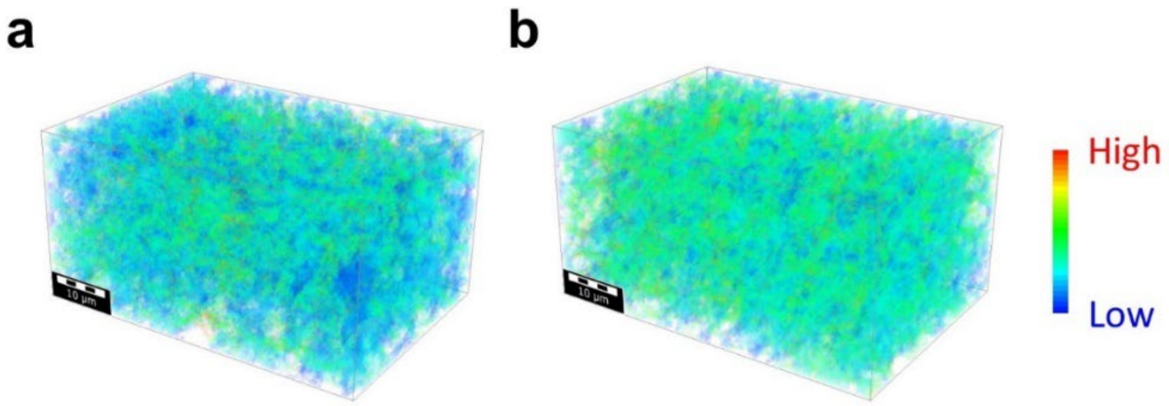

Supplementary Fig. 30. Ionic current density map for a) bare NCM and b) LiDFP NCM.

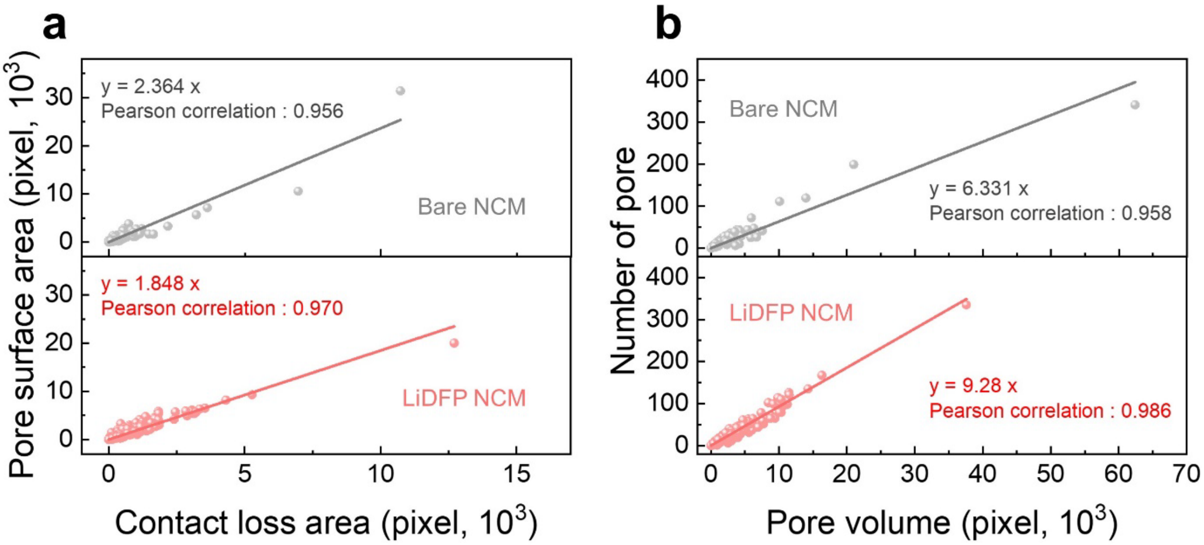

Supplementary Fig. 31. Comparison of a) contact loss area versus pore surface area and b) pore volume versus number of pore for each labeled cathode particle, both bare NCM and LiDFP NCM.

Supplementary Table 5. Symbols and denoted meanings for chemical diffusion coefficient.

**Symbol**	**Meaning**
D^{GITT}	Chemical diffusion coefficient (cm^2/s)
ΔE_s	Steady-state voltage changes due to the current pulse (V)
ΔE_t	Voltage changes during the constant current pulse (V)
S	Active surface area (cm^2)
τ	Duration of the current pulse (s)
n_m	Number of moles (mol)
V_M	Molar volume of the electrode (cm^3/mol)

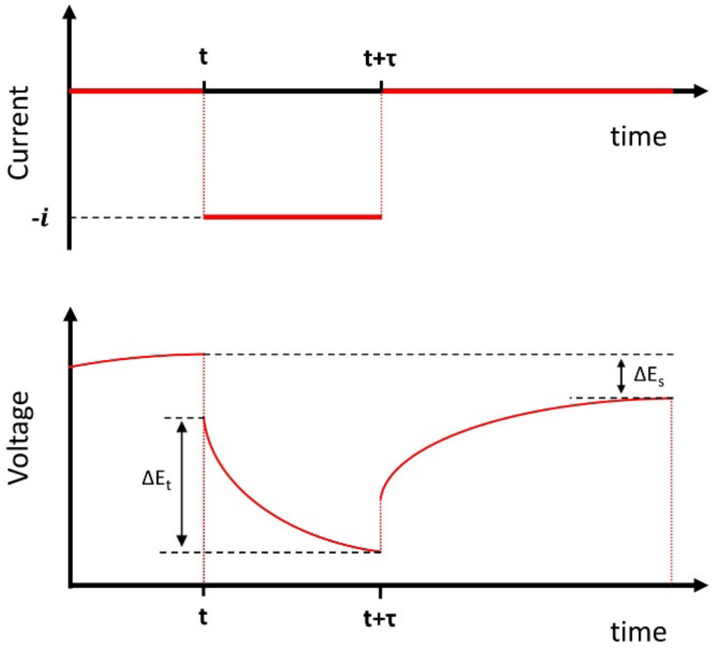

Supplementary Fig. 32. A constant current pulse is imposed for the duration time (τ) at time t, and the resulting voltage changes.

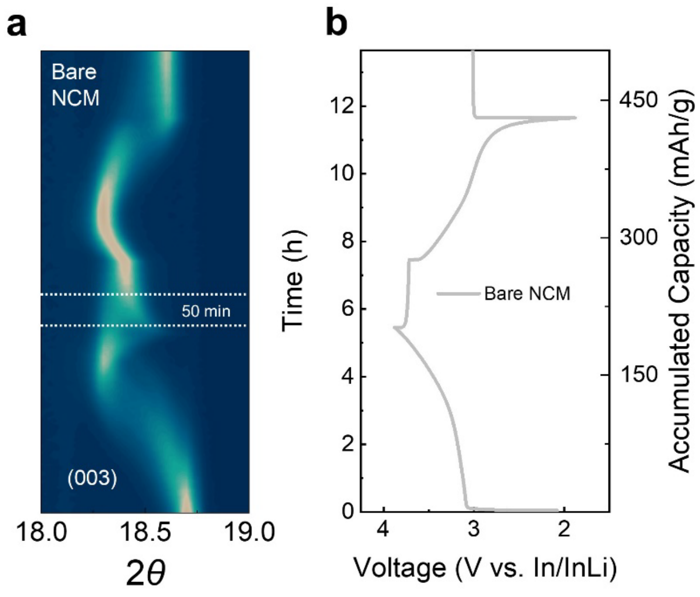

Supplementary Fig. 33. Contour plot of the diffraction peak evolution of the (003) planes for fresh bare NCM with 1h rest after charge and discharge process.

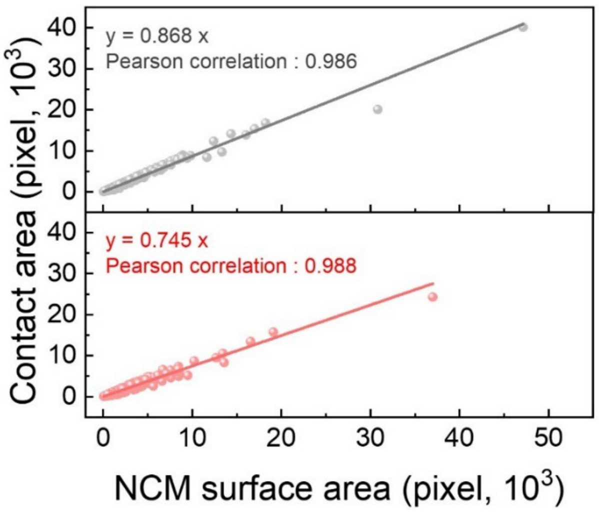

Supplementary Fig. 34. Comparison of NCM surface area versus contact area for both bare NCM and LiDFP NCM.

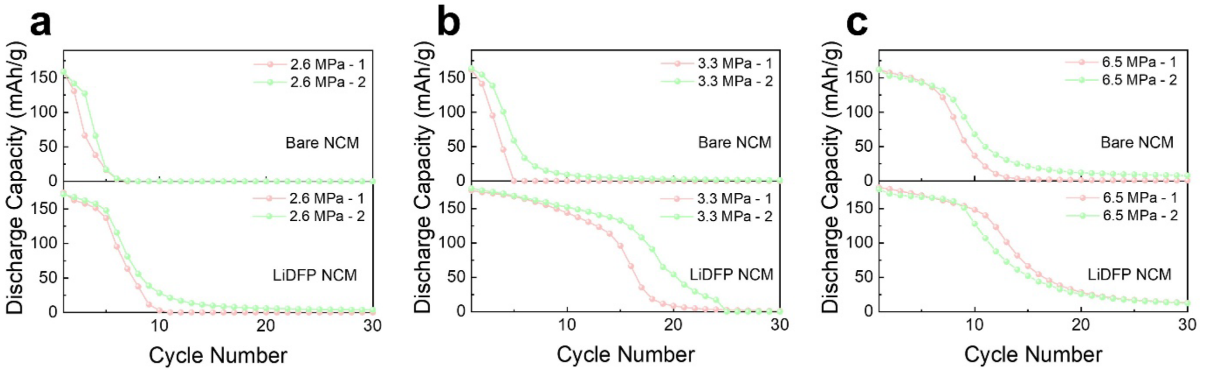

Supplementary Fig. 35. Reproducible electrochemical cycle performance of bare NCM and LiDFP NCM at C/10 (1C = 1.36 mA/cm^2) rate and 25 °C under varying stack pressure of a) 2.6, b) 3.3, and c) 6.5 MPa. Each cell labeled as ‘1’ in the figure corresponds to the cell shown in Fig. 4i.

References

1. Banerjee, A., Wang, X., Fang, C., Wu, E. A. & Meng, Y. S. Interfaces and Interphases in All-Solid-State Batteries with Inorganic Solid Electrolytes. *Chem. Rev.* **120**, 6878–6933 (2020).

2. Wan, T. H., Saccoccio, M., Chen, C. & Ciucci, F. Influence of the Discretization Methods on the Distribution of Relaxation Times Deconvolution: Implementing Radial Basis Functions with DRTtools. *Electrochim. Acta* **184**, 483–499 (2015).

3. Xia, J., Wang, C., Wang, X., Bi, L. & Zhang, Y. A perspective on DRT applications for the analysis of solid oxide cell electrodes. *Electrochim. Acta* **349**, (2020).

4. Xu, H., Yang, S. & Li, B. Pressure Effects and Countermeasures in Solid-State Batteries: A Comprehensive Review. *Adv. Energy Mater.* **14**, 2303539 (2024).

5. Whittingham, M. S. Lithium batteries and cathode materials. *Chem. Rev.* **104**, 4271–4301 (2004).

6. Jo, S. *et al.* Solid-State Reaction Heterogeneity During Calcination of Lithium-Ion Battery Cathode. *Adv. Mater.* **35**, 2207076 (2023).

7. Münch, B., Trtik, P., Marone, F. & Stampanoni, M. Stripe and ring artifact removal with combined wavelet — Fourier filtering. *Opt. Express,* **17**, 8567–8591 (2009).

8. Weng, W. & Zhu, X. U-Net: Convolutional Networks for Biomedical Image Segmentation. *IEEE Access* **9**, 16591–16603 (2015).

9. Min, J., Gubow, L. M., Hargrave, R. J., Siegel, J. B. & Li, Y. Direct measurements of size-independent lithium diffusion and reaction times in individual polycrystalline battery particles. *Energy Environ. Sci.* **16**, 3847–3859 (2023).

10. Wang, D. *et al.* Realizing high-capacity all-solid-state lithium-sulfur batteries using a low-density inorganic solid-state electrolyte. *Nat. Commun.* **14**, 1–10 (2023).
