# Supplementary material for: Interfacial chemistry-driven reaction dynamics and resultant microstructural evolution in lithium-based all-solid-state batteries
Source: Nat Commun. 2025 Oct 3;16:8838. doi: 10.1038/s41467-025-63959-1 (PMC12494980; doi:10.1038/s41467-025-63959-1)
Supplement: Supplementary file 2 — Description of Additional Supplementary Files [file 41467_2025_63959_MOESM2_ESM.pdf]

Supplementary Video 1. Representative 3D images of identification and quantitative information for labeled cathode, contact loss area, and labeled pore of Bare NCM.

Supplementary Video 2. Representative 3D images of identification and quantitative information for labeled cathode, contact loss area, and labeled pore of LiDFP NCM.
